# Implementation of a Mobile Health Strategy to Improve Linkage to and Engagement with HIV Care for People Living with HIV, Tuberculosis, and Substance Use in Irkutsk, Siberia

**DOI:** 10.1089/apc.2020.0233

**Published:** 2021-03-09

**Authors:** Jacqueline Hodges, Svetlana Zhdanova, Olga Koshkina, Alexey Suzdalnitsky, Ava Lena Waldman, Jason Schwendinger, Serhiy Vitko, Alexey Plenskey, Yulia Plotnikova, Elena Moiseeva, Mikhail Koshcheyev, Sergey Sebekin, Oleg Ogarkov, Rebecca Dillingham, Scott Heysell

**Affiliations:** ^1^ Division of Infectious Diseases and International Health, University of Virginia, Charlottesville, Virginia, USA.; ^2^ Department of Epidemiology and Microbiology, Scientific Centre for Family Health and Human Reproduction Problems, Irkutsk, Russian Federation.; ^3^ Irkutsk Regional Tuberculosis Referral Hospital, Irkutsk, Russian Federation.; ^4^ Irkutsk Regional AIDS Centre, Irkutsk, Russian Federation.

**Keywords:** human immunodeficiency virus, acquired immunodeficiency syndrome, tuberculosis, mobile health, linkage to care

## Abstract

In Irkutsk, Siberia, there is a high prevalence of HIV and tuberculosis (TB) coinfection. Mobile health (mHealth) strategies have shown promise for increasing linkage to and engagement in care for people living with HIV (PLWH) in other contexts. We evaluated outcomes for a cohort of PLWH, TB, and substance use in Irkutsk after participation in a multi-feature mHealth intervention called MOCT. Sixty patients were enrolled during hospitalization for TB. We evaluated participant app usage, linkage to HIV care postdischarge, perception of self-efficacy related to HIV care, and HIV-related clinical outcomes at 6 months. We also performed an exploratory analysis to compare a subset of 49 patients with a pre-intervention cohort matched for age and gender. Participants demonstrated engagement with app features examined at 6 months. The majority linked to HIV care by 6 months (83%). Self-scoring of confidence in ability to communicate with HIV providers improved from baseline (median score 8, scale 1–10) to 6 months (10, *p* = 0.004). A higher proportion of the MOCT subset refilled antiretroviral therapy (69% vs. 43% in pre-intervention cohort, *p* = 0.01), with fewer deaths in the MOCT subset at 6 months (1 death vs. 10 deaths in pre-intervention cohort, *p* = 0.02) and a decreased likelihood of developing the composite outcome of death/failure to achieve viral suppression at 6 months (adjusted odds ratio = 0.33, *p* = 0.029). This study demonstrates preliminary intervention uptake and improvement in short-term outcomes for an urban cohort of PLWH, TB, and substance use enrolled in a multi-feature mHealth intervention, a novel strategy for the context.

Clinical Trial Registration Number: NCT03819374.

## Introduction

Within the Russian Federation (RF), the HIV epidemic is still growing despite gains made to slow infections.^1,2^ HIV infection is one of the top 10 causes of premature death in the RF.^3^ People who inject drugs account for a high proportion of new HIV cases,^4,5^ and they experience suboptimal coverage with antiretroviral therapy (ART) after diagnosis,^3,6^ with viral suppression rates as low as 26% in the RF.^7^ The RF also shoulders some of the highest rates of multi-drug-resistant (MDR) tuberculosis (TB). TB accounts for a third of HIV-associated deaths globally.^8^ In Siberia, TB incidence estimates are 1.6 times higher than the national rate.^3^ Within Siberia, Irkutsk is the federal jurisdiction with the highest HIV/TB coinfection prevalence.^9^ In Irkutsk, disengagement with HIV care is a primary driver of HIV/TB morbidity and mortality, which makes early ART initiation a high priority for coinfected patients, and inpatient TB hospitalization serves as a critical opportunity for engagement^10,11^

Mobile health (mHealth) strategies to improve linkage to and retention in HIV care and optimize adherence to ART have shown promise in diverse settings.^12–15^ mHealth strategies trialed in other HIV/TB-prevalent contexts to improve ART initiation and retention in care for patients with TB have employed single-feature mobile phone interventions. These have yet to demonstrate an improvement in these outcomes and, to our knowledge, they have not been designed for the Russian context.^16,17^ Our group has previously developed a smartphone-based intervention called PositiveLinks for a rural population of people living with HIV (PLWH) in the United States. PositiveLinks implementation was associated with improvement in several clinical outcomes.^12^ We hypothesized that, given relative smartphone familiarity and similar barriers of geographic remoteness existing in Irkutsk, a culturally tailored mHealth platform could improve linkage to HIV care for patients with comorbid TB and a history of substance use. We aimed to evaluate patient usage of the mHealth intervention, linkage to HIV care, and self-efficacy relative to HIV care in the 6 months after enrollment in the intervention. We also compared clinical outcomes for a subset of this study's cohort with those of a cohort of PLWH with a history of using substances that were hospitalized for inpatient TB therapy immediately before intervention implementation.

## Methods

### Setting and study design

Patients hospitalized at the Irkutsk Regional Tuberculosis Referral Hospital (TB Referral Hospital) were consecutively enrolled from April 2018 to November 2019 in the mHealth intervention (“MOCT”—Russian for “bridge”). Follow-up TB care was delivered at the TB Referral Hospital or affiliated TB clinics, and follow-up HIV care at the Irkutsk Regional AIDS Centre (AIDS Centre). Inclusion criteria included adults 18–64 years of age, with HIV status confirmed by chart review or laboratory testing, a history of using substances upon recruitment (confirmed by chart review or self-report), and primarily residing in Irkutsk city at the time of recruitment. Patients unable or unwilling to use a smartphone or cognitively unable to give informed consent were excluded. Written informed consent was obtained for all participants based on a protocol approved by the institutional review boards at the Scientific Centre for Family Health and Human Reproduction Problems (RF) and the University of Virginia (USA). This study used a single-arm prospective design with standardized assessments at baseline, 2 months, and 6 months.

### The MOCT app intervention

**FIG. 1.**
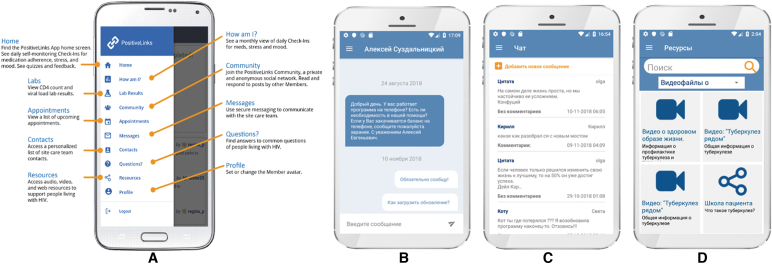
MOCT platform features. PositiveLinks sidebar with full description of features **(A)**. MOCT platform features include a direct messaging feature that allows patients to communicate with clinic care team members **(B)**, a community message board for anonymous peer messaging **(C)**, tailored educational resources for HIV, TB, and substance use **(D)**, daily queries of stress, mood, and TB and HIV medication adherence, appointment reminders, and access to TB and HIV laboratory results. TB, tuberculosis. (Color image can be found at www.liebertonline.com/apc).

The MOCT smartphone app was adapted from PositiveLinks, an app originally developed using a theory-based iterative user-centered process.^12^ After translation and management of logistical considerations for implementation within Irkutsk, including server management, security, and privacy, we elicited TB Referral Hospital and AIDS Centre provider and patient feedback using unstructured interviews regarding platform design and functionality, usability, and acceptability. The MOCT platform provides several features as described in Fig. 1. Clinic staff were appointed to serve as app administrators responsible for troubleshooting user difficulties, moderating, and responding to user content. Patients were trained on the functionalities of the app, including a short proficiency test before downloading the app. Patients who did not own a smartphone were issued one along with a data plan for the duration of the study period.

### Data collection

#### Baseline and clinical laboratory value assessment

Baseline characteristics were collected upon enrollment using structured patient interviews and chart review. Data for laboratory values including CD4 count (cells/mm^3^) and HIV viral load (VL, copies/mL), and ART refills obtained from AIDS Centre pharmacy records were collected within 90 days before the baseline time point and within 90 days before or after the 6-month time point.

#### Outcomes

##### MOCT app engagement

User engagement with app features was automatically tracked by the app and downloaded from the web-based administrator portal. This included responses to daily queries, direct provider messaging, and community message board posts.

##### Patient linkage to and self-efficacy in HIV care

Data relating to patient linkage (or re-linkage) to HIV care and self-efficacy relative to HIV care was collected at baseline, 2-, and 6-month assessments. A patient was considered “linked to care” at the 2- or 6-month time point if he or she reported attending at least one AIDS Centre appointment by that assessment. Loss to follow-up was defined as no patient registration information available in the AIDS Centre electronic medical record system after enrollment. Patient self-efficacy relative to retention in HIV care was evaluated using an adapted and translated 14-item retention-in-care assessment tool^18^ that allows patients to rate specific measures (on a 1–10 point Likert scale).

### Exploratory comparison with pre-intervention cohort

The same study inclusion criteria were applied to the pre-intervention cohort, including documentation of presumed ability to give informed consent. Baseline characteristics were collected by chart review. Time points for the pre-intervention cohort were defined relative to the date of discharge from the TB Referral Hospital. A post hoc exploratory analysis was performed to compare 6-month outcomes for a subset of participants in the MOCT intervention with a historical (pre-intervention) cohort of PLWH with a history of substance use who were hospitalized at the TB Referral Hospital in the year before the start of enrollment. No other significant practice changes were identified in the pre-intervention and MOCT intervention time periods. Exploratory variables of interest for comparison between cohorts by 6 months included viral suppression and death (all causes) obtained by chart review, as well as dispenses of at least one refill of ART obtained by pharmacy monitoring.

### Statistical analyses

Baseline characteristics, linkage to care, and app feature usage were analyzed using descriptive statistics. A subset of patients in the pre-intervention and MOCT cohorts were matched using random 1:1 matching with a tolerance of 5 years for age and exact matching for gender. A total of 49 matches were obtained from a pool of 50 patients with sufficient data on chart review during the pre-intervention period to meet study inclusion criteria. The Wilcoxon signed-rank test was used to compare continuous variables for the MOCT cohort between baseline and 6 months (retention-in-care scale score, CD4 count), and between the matched pre-intervention and MOCT cohorts at baseline (CD4 count, VL). McNemar's test was used to compare dichotomous variables for the MOCT cohort between time points (viral suppression, users of app features), and between matched cohorts at baseline [CD4 count <200, viral suppression, MDR TB/rifampin resistance, extensively drug-resistant (XDR) TB] and at 6 months (proportion of patients with viral suppression, at least one ART refill dispensed, and deceased). Binomial logistic regression was performed to examine the association between the composite outcome of death/failure to achieve viral suppression at 6 months with covariates, including exposure to the MOCT intervention. Analyses were performed with IBM SPSS Statistics for Mac, Version 26.0 (Statistical Package for the Social Sciences, IBM Corp, Armonk, NY).

## Results

### Baseline characteristics

Sixty patients were enrolled in the intervention. Majority were male (60%) with a mean age of 38 years (Table 1). Chart review indicated that 33% of patients enrolled in the intervention were on ART immediately before admission to the TB Referral Hospital, whereas 26 additional patients (43%) reported receiving ART at some point before hospitalization. Viral suppression was low at baseline (27%), and only 20% of those on ART immediately before admission were virally suppressed at baseline. A significant proportion of the cohort had a baseline CD4 count <200 (45%) and drug-resistant TB (XDR or MDR, 44%). The most common agents included in the ART regimens used in this study were lamivudine (78%), tenofovir (58%), and efavirenz (47%).

**Table 1.** Baseline Demographics (*N* = 60)

Characteristic	*N* or mean (% or SD)
Age	38 (6)
Gender, female	24 (40%)
Baseline CD4 count (cells/mm^3^)	233 (207)
Baseline CD4 count <200	27 (45%)
Baseline viral load (copies/mL)	87,142 (276,976)
Baseline viral suppression	16 (27%)
On antiretroviral therapy before hospitalization	20 (33%)
Chronic hepatitis C infection	26 (43%)
Drug-resistant TB, current episode	26 (43%)
Past TB treatment	14 (23%)
Past drug-resistant TB treatment	5 (8%)
Housing	
Own home	35 (58%)
Family home	18 (30%)
Homeless	4 (7%)
Other	2 (3%)
History of incarceration	14 (23%)
Monthly income (rubles)	26,268 (17,439)
Substance use history	
Any intravenous drug use	32 (53%)
Any drugs smoked	25 (42%)
Opiates/opioids	14 (23%)
Methamphetamine	0 (0%)
Cocaine	0 (0%)
Alcohol (any)	50 (83%)
Alcohol (most days/to intoxication)	19 (32%)
Marijuana	22 (37%)

Baseline viral suppression defined as VL <200 copies/mL. Drug-resistant TB includes MDR/rifampin resistant TB, defined as rifampin resistance on Gene Xpert testing or other phenotypic testing, and XDR TB, or additional resistance to at least one fluoroquinolone and a second-line injectable drug (amikacin, capreomycin, or kanamycin).

MDR, multi-drug-resistant; TB, tuberculosis; VL, viral load; XDR, extensively drug-resistant.

### MOCT app engagement

Aggregate cohort usage of each app feature examined is summarized (Table 2). By 6 months after enrollment, 33 patients (55%) sent at least one message, 34 (57%) posted on the community message board at least once, and 43 (72%) responded to at least one daily query. Forty-three patients (72%) used at least one of the features examined, and 51 (85%) logged in to the app at least once during the 6-month period. Compared with the first month, the messaging feature demonstrated the smallest drop in active users (participants using the feature at least once that month) by 6 months (21 vs. 15, *p* = 0.18). However, there was a significant difference in the proportion of active users for the community message board (23 vs. 8, *p* = 0.003) and daily queries (41 vs. 19, *p* < 0.001) between those time points. No security breaches of the portal occurred.

**Table 2.** MOCT App User Activity Summary

	Month 1	Month 2	Month 3	Month 4	Month 5	Month 6
Direct messages						
Total sent by cohort, *N*	178	156	193	149	121	108
Sent per user, mean (SD, range)	3.2 (7.6, 0–39)	2.8 (8.5, 0–55)	3.5 (9.4, 0–60)	2.7 (8.5, 0–56)	2.3 (5.9, 0–34)	1.9 (5.3, 0–30)
Active users, *N*	21	15	15	12	14	15
Community message board posts						
Total sent by cohort, *N*	75	27	21	16	26	21
Sent per user, mean (SD, range)	1.3 (2.8, 0–12)	0.5 (1.7, 0–9)	0.4 (1.5, 0–10)	0.3 (0.9, 0–5)	0.5 (1.1, 0–6)	0.4 (1.2, 0–7)
Active users, *N*	23	7	7	8	13	8
Daily query responses						
Monthly response rate, mean, % (SD)	43% (39%)	32% (40%)	28% (39%)	27% (37%)	25% (39%)	24% (38%)
Monthly response rate, mean, active users only % (SD)	59% (34%)	64% (35%)	69% (31%)	62% (31%)	68% (35%)	74% (27%)
Active users, *N*	41	28	25	24	21	19
Any of the features examined						
Active users, *N*	41	29	26	25	21	20

Feature usage by month after patient enrollment. Active users defined as participants using the respective feature at least once during the corresponding month postenrollment.

### Patient linkage to and self-efficacy in HIV care

Survey responses were missing for 10 patients at 6 months due to loss to follow-up (7), death (2), and no-show to 6-month assessment but ongoing AIDS Centre follow-up (1). Six of the seven patients lost to follow-up eventually re-established or linked to care after the end of the study assessment period. At 2 months, 40 patients reported linkage to care, or attending at least one visit since their baseline assessment. Thirty-six patients reported attending at least one additional visit by their 6-month assessment. Ten patients reported attending at least one visit for the first time at the 6-month assessment, for a total of 50 patients linked to care by 6 months (83%).

Analysis of retention-in-care scale scores at 6 months (*N* = 50 surveys with responses available) compared with baseline demonstrated that patients' median scoring of confidence in their ability to regularly “contact the clinic if I have questions about my medications” improved (Table 3, *p* = 0.004). The median score on patient confidence in setting health goals was high at baseline with a wider spread in responses at 6 months.

**Table 3.** Retention-in-Care Scale

Scale item	Median score [IQR], baseline	Median score [IQR], 6 months	*p*
For each of the following questions, please rate how confident you are that you can regularly perform each task at the present time:			
Make appointment at the clinic	10 [8–10]	10 [8–10]	0.42
Plan transportation to the clinic	10 [8–10]	10 [8.75–10]	0.37
Attend a clinic appointment	10 [8–10]	10 [9–10]	0.51
Understand my CD4 laboratory result	9 [4–10]	9 [5–10]	0.59
Understand my HIV viral load result	9 [4–10]	9 [5–10]	0.17
Pick up medications from the pharmacy	10 [9–10]	10 [9–10]	0.83
Take my medications everyday	10 [9–10]	10 [8–10]	0.48
Contact the clinic if I have questions about my medications	8 [6–10]	10 [9–10]	0.004
Ask questions about HIV to health care providers	10 [8–10]	10 [8–10]	0.41
Tell a loved one that I am living with HIV	10 [5–10]	10 [5–10]	0.73
Tell a romantic partner that I am living with HIV	9 [4–10]	8 [1–10]	0.17
Recognize that I am stressed or sad	10 [8–10]	9 [7–10]	0.33
Identify ways to help me cope if I am stressed or sad	9 [6–10]	9 [5.75–10]	0.36
Set goals for my health	10 [10–10]	10 [7–10]	0.01

Patients rate items on a scale of 1–10 (1 = not confident at all, 10 = totally confident) during baseline and 6-month assessments. Median scores were calculated for those with survey data available at 6 months (*N* = 50), and compared between time points.

IQR, interquartile range.

### CD4 count and viral suppression

Laboratory data were missing for 10 patients at 6 months as noted earlier. The median CD4 count improved at 6 months [274 (interquartile range 139–429, compared with baseline)] [184 (65–335), *p* = 0.048]. The proportion of patients achieving viral suppression also improved at 6 months (*N* = 33 of 50 patients with data available, 55% of cohort) compared with baseline (*N* = 16, 27%; *p* < 0.001).

### Clinical outcomes compared with pre-intervention cohort

Exploratory analysis was performed for a subset of patients in the MOCT cohort matched to the pre-intervention cohort (*N* = 49). The cohorts demonstrated similar baseline characteristics after matching (Table 4). Analysis revealed lower mortality by 6 months in the MOCT subset (1 death, not attributed to HIV or TB) compared with the pre-intervention cohort (10 deaths, 7 attributed to HIV, *p* = 0.02). The monitored dispense rate for ART at 6 months for the MOCT subset (34/49, 69% with at least one refill) was higher than the rate observed for the pre-intervention cohort (21/49 or 43%, *p* = 0.01).

**Table 4.** Comparison of Cohort Baseline Demographics

Demographics	Pre-intervention cohort	MOCT-intervention cohort	*p*
Age, mean (SD)	37 (5)	38 (6)	Matched
Gender, female, *N* (%)	14 (29%)	14 (29%)	Matched
Baseline CD4 count, mean (SD)	192 (224)	209 (163)	0.18
Baseline CD4 count <200, *N* (%)	35 (71%)	22 (45%)	0.052
Baseline VL, mean (SD)	495987 (1437305)	97217 (300709)	0.002
Baseline viral suppression, *N* (%)	11 (22%)	13 (27%)	0.42
MDR/RR TB, *N* (%)	18 (37%)	22 (45%)	0.57
XDR TB, *N* (%)	7 (14%)	1 (2%)	0.07

Cohorts matched by age (within 5 years) and gender (*N* = 49). Viral suppression defined as VL <200 copies/mL. MDR/RR TB defined as rifampin resistance on Gene Xpert testing or other phenotypic testing for current episode. XDR TB defined as additional resistance to at least one fluoroquinolone and a second-line injectable drug (amikacin, capreomycin, or kanamycin).

RR, rifampin resistant.

Binomial logistic regression evaluated the effects of covariates on the likelihood that patients develop the composite outcome of death/unable to achieve viral suppression at 6 months (Table 5). Exposure to the MOCT intervention was associated with a decreased likelihood of developing the composite outcome (adjusted odds ratio = 0.33, *p* = 0.029). Whereas a baseline VL >200 was also independently associated with an increased likelihood for developing the composite outcome (odds ratio = 8.53, *p* = 0.002), associations with the other covariates were not statistically significant.

**Table 5.** Binomial Logistic Regression for Composite Outcome at 6 Months

Covariate	Adjusted odds ratio [95% CI]	*p*
Baseline CD4 > 200	0.78 [0.27–2.23]	0.64
Baseline VL >200	8.53 [2.14–34.02]	0.002
Drug-resistant TB	1.48 [0.55–3.95]	0.44
MOCT app intervention	0.33 [0.12–0.89]	0.029

Composite outcome was defined as death or failure to achieve viral suppression (VL >200) at 6 months. Analysis was performed on the MOCT subset and pre-intervention cohort, matched for age (within 5 years) and gender (*N* = 49). Drug-resistant TB includes MDR/RR or XDR TB.

## Discussion

This study is the first, to our knowledge, to evaluate a multi-feature mHealth strategy among HIV/TB coinfected patients in the RF with substance use and otherwise at high risk of disengagement. The MOCT app was usable based on preliminary analysis, and participants demonstrated improved linkage to and engagement with HIV care as well as improved 6-month clinical outcomes relative to pre-intervention controls.

The cohort continued to use the MOCT app after enrollment. Although retention-in-care scale scores at baseline and 6 months indicated high confidence for patients on all measures, there was a significant increase in confidence related to ability to regularly reach out to providers at the clinic with questions about medications, a feature directly supported by the MOCT app. Messaging activity was highest within the first and third months after enrollment, with consistent use at 6 months. Messaging can enhance patient–provider rapport, and has been shown to be important for maintaining retention in care.^19,20^ Notably, although the number of active users remained relatively constant across the 6 months for the direct messaging feature, there was a significant early drop in the number of active users of the community message board and a more gradual drop in active users of daily queries over 6 months. Reasons for this drop in usage will be explored in further evaluation of app usability and acceptability.

Good rates of patient linkage to HIV care were observed by 6 months. The intervention provided patients with the opportunity to engage with app features while hospitalized at the TB Referral Hospital and after discharge, when patients needed to coordinate their HIV and TB care independently. A recent systematic review described the heterogeneity of studies of SMS-based mHealth interventions for improving linkage and retention in care for PLWH in low- and middle-income countries and demonstrated in many settings a lack of clear benefit.^21^ In contrast to mobile phone interventions delivered as stand-alone strategies, the multi-feature MOCT app was intentionally embedded in a health systems integration effort for coordination between the TB Referral Hospital and the AIDS Centre through weekly meetings of institution representatives, shared access to laboratory values, medication refill data, and appointment scheduling. The significantly improved mean CD4 count and rates of viral suppression at 6 months were likely the effect of enhanced adherence in patients already on ART before enrollment as well as an increase in ART coverage for patients who were not previously prescribed ART. Examination of these outcomes at the 6-month time point limits the ability to detect maximal viral suppression, as the most commonly used ART regimens in this study are associated with longer time to suppression relative to the integrase inhibitors now in more common use in the RF.

Exploratory analysis of the matched cohorts revealed a relative reduction in 6-month mortality in the MOCT cohort. There was also a statistically significant decrease in the likelihood for development of the composite outcome at 6 months for the MOCT cohort when controlling for other covariates related to severity of TB and HIV at baseline. We were unable to control for ongoing substance use patterns as these were not measured in the pre-intervention cohort. Early ART initiation followed by ongoing adherence is associated with reduced mortality in patients with HIV/TB coinfection,^22^ and mortality typically occurs early during the anti-TB treatment course for coinfected patients not on ART.^23^ We observed higher rates of ART refills for the MOCT cohort by 6 months, and the cause of death at 6 months showed the majority were attributed to HIV in the pre-intervention cohort, whereas the single death in the MOCT subset was not related to HIV or TB.

Limitations exist for this study. Loss to follow-up resulted in missing 6-month data on linkage to care and self-efficacy. The loss of these participants may introduce attrition bias, inflating the proportion of favorable survey responses obtained for retention-in-care scale scoring at 6 months. It also excludes laboratory values for patients unlikely to be virally suppressed at 6 months. Direct translation of several retention-in-care scale items during initial testing proved incomprehensible in Russian and required modification, which may have had unintended consequences on how patients ultimately responded to the scale. Twenty-five participants were provided a smartphone, and all were provided data plans, which may have motivated some patients to demonstrate app usage during ongoing assessments, but usage was not stated as a requirement to keep the phone. Deploying the platform through a smartphone may also limit the generalizability of findings if smartphones remain out of reach for PLWH and TB in other high-burden regions.

The comparison between a pre- and MOCT-intervention cohort was not randomized to the intervention/standard of care or matched by baseline disease characteristics. Instead, we had to adjust for those characteristics by building them into our regression analysis. The comparative analysis was performed for two relatively small sample populations selected from a regional referral hospital specializing in the care of people with advanced disease and there may have been a higher underlying propensity for patients to encounter HIV/TB treatment failure that was not directly accounted for in our analysis. An additional residual confounder that may contribute to the observed improvement in outcomes for the MOCT cohort includes higher frequency of provider-initiated contact with patients associated with conducting study activities. The matching process also introduces some potential bias. The majority of the MOCT cohort participants excluded from exploratory comparison (9/11) were female; however, rates of 6-month mortality (1 death) and viral suppression (7/11, 64%) were not substantially different from those observed for the MOCT subset after matching.

In conclusion, the MOCT app intervention employed a multi-feature mHealth strategy that is novel for the treatment of HIV in this context. There was sustained engagement in HIV care for a majority of the enrolled cohort by 6 months. Further work will study the scalability of the MOCT intervention after further iteration, including how the app may specifically enhance integration of care for PLWH/TB, and whether similarly positive outcomes are found if applied to PLWH without TB, or those already in the outpatient setting.
